# Transforming research in team‐based learning

**DOI:** 10.1111/medu.70128

**Published:** 2025-11-28

**Authors:** Tracey Edelist, Stella Ng

**Affiliations:** ^1^ CACHE, University Health Network University of Toronto Toronto Canada

## Abstract

Team‐based learning is an opportunity for students to learn about one another, engaging in the ideals of both interprofessional and transformative education.

Cleland, Pienkowska, and Kitto's scoping review of research on the pedagogical approach of team‐based learning (TBL) in medical education confirmed their supposition that there has been little research to expand knowledge of the field since TBL's incorporation into medical education in the 2000s. TBL has become an accepted pedagogy with little critical assessment of how it works and how it could work better. The review revealed few exploratory studies compared to those assessing feasibility, acceptability, engagement and knowledge outcomes.^1^ To improve the quality of TBL, the authors argue research should shift to future development rather than continuing to focus on what is already known about TBL implementation. We agree that studying TBL as a given, rather than examining and expanding our mechanistic understanding, ultimately limits its potential impact. As researchers of constructivist, cognitivist, and critical pedagogies^2^ situated in collaborative practice environments, we offer additional lines of inquiry for further study: team composition, transformative interprofessional education and evidence‐informed teaching and learning approaches.

Within team‐based practice, team context, dynamics and engagement greatly impact the possibilities for respectful, open, honest communication between team members,^3^ yet the current review's findings demonstrate very few TBL studies considered these factors. TBL does focus on developing teams balanced in terms of the diversity of students' characteristics relevant to each institution's context and students' practical and academic background knowledge.^4^ However, we agree it would be fruitful to learn more about how team composition and dynamics affect both the process and outcomes of TBL. In addition, rather than taking for granted the affordances of learning within a team, TBL literature ought to delve deeply into the requisite knowledge, capability and resources that may support optimal team learning experiences and outcomes.

The authors question the usefulness of uni‐disciplinary TBL considering the multi‐disciplinary nature of healthcare. We support further research applying pedagogies used within interprofessional education (IPE), and other pedagogies suited for collaborative learning between disciplines, to inter‐disciplinary TBL. In bringing together small groups of students to learn from and with one another, we believe TBL provides an additional opportunity for students to learn *about* one another, engaging in the ideals of both interprofessional and transformative education. IPE does not necessarily engage with the formal construct of TBL; however, it has the necessary ingredients to do so given the small group emphasis. In IPE, the ethos is to learn with, from and about diverse team members and disciplines. In transformative education, learning encompasses knowledge acquisition while also questioning individual and societal assumptions, working towards a goal of equitable healthcare.^2^, ^5^

We believe TBL provides an additional opportunity for students to learn about one another, engaging in the ideals of both interprofessional and transformative education.

In a transformative interprofessional approach to TBL, students could be encouraged to build on concrete concepts learned (e.g. biomedical content) by questioning implicit assumptions and power relations that may limit the relevance of formal knowledge in contexts excluded in the creation of such knowledge. In this vein, TBL could be studied for its relevance to teaching about health equity, as well as whether TBL can support a process of learning that is itself equitable and critically reflective. For example, the process of TBL could be used to explicitly teach students how to engage in challenging yet constructive dialogue, respectfully considering opposing opinions to determine solutions together.^6^

The process of TBL could be used to explicitly teach students how to engage in challenging yet constructive dialogue.

These proposed transformative and interprofessional approaches to TBL would require further research on TBL learning outcomes. The types of outcomes typically measured in TBL relate to individual and team readiness assurance tests (IRA/TRA) and exam scores. If learners were prompted to question knowledge sources and the way power relations impact their thinking, assessment would also need to align with these broader purposes.^2^, ^7^ For example, aligned approaches may explore how to assess capabilities and processes evidenced by the way students talk, interact and write (e.g. assessing for growth in how they invite and consider different perspectives).^8^

TBL is described as a pedagogical approach, whereas the lack of pedagogical inquiry related to TBL leads us to wonder if it may be better described as an overarching educational design into which evidence‐informed pedagogies can then be applied.^9^ ‘We know little about how the space in which TBL occurs may influence teaching and learning, or about the role of the teacher in the TBL process’.^1^ In other words, instructors must still engage with other pedagogies to teach material to students; TBL does not provide guidance about *how* to teach; it only provides the structure within which to teach and learn. This opening welcomes a wide variety of educational approaches with strong evidence bases (e.g. Baker et al^2^) and thus necessitates a more systematic approach to pedagogical and instructional design research.

TBL does not provide guidance about how to teach; it only provides the structure within which to teach and learn.

Finally, we note that the findings of this scoping review align with traditional priorities and philosophies for education in medicine. The findings of the review demonstrate TBL's alignment with medical hierarchies, and, as the authors note, position TBL as a means to an end (better scores), rather than examining how TBL itself can influence learners in ways not easily captured by surveys and scores. TBL, despite being team‐based, could inadvertently support a culture of competition, hierarchies and privileging certain knowledge held by specific people/experts over others.^10^, ^11^ This possibility is highlighted by the little attention within TBL research given to team dynamics, context, relationships, what people learn from one another, and what information is taught to the team and how.

TBL, despite being team‐based, could inadvertently support a culture of competition, hierarchies and privileging certain knowledge held by specific people/experts over others.

We appreciate the points the authors raise about the lack of critical examination of TBL within medical education and agree future research needs to address those concerns. In particular, we hope for research on team development and process, in‐depth examination of the mechanisms underpinning TBL and evidence‐informed and theoretically aligned teaching, learning and assessment approaches that could be integrated into TBL to enhance its effectiveness across contexts.

## CONFLICT OF INTEREST STATEMENT

There were no conflicts of interest.

## Data Availability

Data sharing not applicable to this article as no datasets were generated or analysed during the current study.
